# Targeted Dorsal Dentate Gyrus or Whole Brain Irradiation in Juvenile Mice Differently Affects Spatial Memory and Adult Hippocampal Neurogenesis

**DOI:** 10.3390/biology10030192

**Published:** 2021-03-04

**Authors:** Céline Serrano, Morgane Dos Santos, Dimitri Kereselidze, Louison Beugnies, Philippe Lestaevel, Roseline Poirier, Christelle Durand

**Affiliations:** 1 Laboratory of Experimental Radiotoxicology and Radiobiology (LRTOX), Research Department on the Biological and Health Effects of Ionizing Radiation (SESANE), Institute for Radiological Protection and Nuclear Safety (IRSN), 92260 Fontenay-aux-Roses, France; celineserranoanne@gmail.com (C.S.); dimitri.kereselidze@cea.fr (D.K.); beugnies.l@gmail.com (L.B.); philippe.lestaevel@irsn.fr (P.L.); 2 Laboratory of Radiobiology of Accidental Exposure (LRAcc), Research Department in Radiobiology and Regenerative Medicine (SERAMED), Institute for Radiological Protection and Nuclear Safety (IRSN), 92260 Fontenay-aux-Roses, France; morgane.dossantos@irsn.fr; 3 Paris-Saclay Neuroscience Institute (Neuro-PSI), University Paris-Saclay, UMR 9197 CNRS, F-91405 Orsay, France

**Keywords:** dorsal dentate gyrus, adult hippocampal neurogenesis, spatial memory, postnatal irradiation

## Abstract

**Simple Summary:**

The effects of exposure of the juvenile brain to doses of ionizing radiation (IR) ≤ 2 Gy on cognitive functions in adulthood are not clearly established in humans, and experimental data are scarce. To elucidate how IR can impact the postnatal brain, we evaluated and compared the effect of whole brain (WB) or hippocampal dorsal dentate gyrus (DDG) X-ray exposure (0.25–2 Gy) on spatial memory, three months after irradiation in mice. In our dose-ranging study, spatial memory was not modified after WB exposure, whereas a deficit was highlighted when irradiation beams were focused on the DDG at the dose of 1 Gy, but not for the lowest or highest doses tested. At 1 Gy, DDG irradiation appeared to be more deleterious to spatial memory and also to adult hippocampal neurogenesis than WB irradiation. Alterations in the generation of newborn neuronal cells in the DG may participate in the memory impairment observed after DDG irradiation at this dose. Finally, our work shows that the brain’s response to IR is complex and depends on the dose and the irradiated brain volume. The societal interest of this study is notably linked to the advent of computed tomography scans for head exploration in children.

**Abstract:**

The cognitive consequences of postnatal brain exposure to ionizing radiation (IR) at low to moderate doses in the adult are not fully established. Because of the advent of pediatric computed tomography scans used for head exploration, improving our knowledge of these effects represents a major scientific challenge. To evaluate how IR may affect the developing brain, models of either whole brain (WB) or targeted dorsal dentate gyrus (DDG) irradiation in C57Bl/6J ten-day-old male mice were previously developed. Here, using these models, we assessed and compared the effect of IR (doses range: 0.25–2 Gy) on long-term spatial memory in adulthood using a spatial water maze task. We then evaluated the effects of IR exposure on adult hippocampal neurogenesis, a form of plasticity involved in spatial memory. Three months after WB exposure, none of the doses resulted in spatial memory impairment. In contrast, a deficit in memory retrieval was identified after DDG exposure for the dose of 1 Gy only, highlighting a non-monotonic dose-effect relationship in this model. At this dose, a brain irradiated volume effect was also observed when studying adult hippocampal neurogenesis in the two models. In particular, only DDG exposure caused alteration in cell differentiation. The most deleterious effect observed in adult hippocampal neurogenesis after targeted DDG exposure at 1 Gy may contribute to the memory retrieval deficit in this model. Altogether these results highlight the complexity of IR mechanisms in the brain that can lead or not to cognitive disorders and provide new knowledge of interest for the radiation protection of children.

## 1. Introduction

Whilst it is apparent that juvenile brain exposure to high doses of ionizing radiation (IR) can lead to neurocognitive toxicity, including memory impairments, lower intellectual quotient and reduced processing speed, several years later in pediatric cancer survivors [1,2] (for review, see [3]), the effects of lower doses of IR (≤2 Gy) on the developing brain are not clearly established (for review, see [4]). Assessment of cognitive functions many years after computed tomography (CT) scans (X-rays: 30–50 mGy) exploration in children 11 years of age revealed no impairment [5,6]. However, another study suggested some cognitive defects, testing learning ability and logical reasoning, in young adults whose brain had been exposed, in a therapeutic context, at doses of IR greater than a few hundred mGy when they were a few months old [7]. Finally, the sparse available data are partially controversial [8] and these studies can present some bias, notably in recruitment or exposure assessment (for review, see [4]). Thus, the literature data provide no clear conclusion regarding the absence of potential long-term adverse effects of these types of IR exposure and highlight the crucial need to improve our scientific knowledge in this field, in particular, because millions of CT scans are practiced worldwide each year, including children (for review, see [9]), a sensitive population with a long life expectancy, mainly for head exploration [10,11,12].

To date, few experimental studies have assessed the long-term effects of postnatal exposure to IR at doses ≤ 2 Gy on cognitive functions and most have used whole body exposure [13,14,15,16]. Thus, when behavioral alterations were observed, it was difficult to assess whether these were exclusively related to the direct effects of IR on the brain. Concerning behavioral effects after postnatal brain irradiation, a severe lack of data can be underlined. We have recently shown higher anxiety/depressive-like behaviors in adult mice after whole brain (WB) exposure, on postnatal day 10 (PND 10; IR ≤ 1 Gy; [17]). In contrast, concerning cognitive functions, Casciati et al. (2016) found no spatial memory deficit in adult mice postnatally exposed to WB IR (PND 10; 0.1 and 2 Gy; [18]). In this context, it appears to be a priority to further study the direct effects of low to moderate doses (≤2 Gy) of IR on the postnatal developing brain and the underlying mechanisms leading to potent radiation-induced cognitive defects [19].

Among the brain structures involved in cognition, the hippocampus (HPC) plays a major role in the processing and storage of different types of memories, notably spatial memory in the case of its posterior (or dorsal in rodents) part (for a review, see [20]). From studies based on high doses of IR, it is known that the HPC and its associated cognitive functions are particularly sensitive to IR [21,22,23,24]. This higher sensitivity may be due to a disturbance of postnatal/adult neurogenesis that continues throughout life in the dentate gyrus (DG) of the HPC [25]. With low to moderate doses of IR (≤2 Gy), several experimental findings illustrate alterations of this dynamic process [15,18], ranging from the proliferation of neural precursors to synaptic integration of newborn neurons after different steps of differentiation and maturation [26]. At present, there is strong evidence indicating that alterations in adult hippocampal neurogenesis contribute to cognitive deficits, including in spatial memory (for reviews, see [27,28,29]).

In the present study, we aimed to evaluate and compare the direct effects of X-rays (≤2 Gy) on the postnatal brain and on the dorsal DG by assessing spatial memory in adult mice and by analyzing different steps of adult hippocampal neurogenesis. To achieve this goal, we performed a dose-response study (0.25–2 Gy) using our two previously developed irradiation models in ten-day-old mice: a model of WB exposure and a model of targeted dorsal dentate gyrus (DDG) exposure, in which the ballistic specificity has been validated using γ-H2AX staining [17]. We chose to irradiate mice at 10 days of age, during the peak of the brain growth spurt, when the immature brain is heavily remodeled [30]. This age is also known to be in a critical sensitive period for toxic agents [16,31]. For the behavioral task, we selected a spatial learning massed protocol in a water maze that is known to involve the activation of adult-born dentate granule cells [32,33,34]. Thus, we showed that postnatal WB exposure to IR did not disturb long-term spatial memory in adult mice, whatever the dose. Nevertheless, when the irradiated zone was focused on the DDG, mice displayed memory deficit three months after postnatal irradiation at 1 Gy. Surprisingly, this cognitive process was not impacted when the applied irradiation dose was 2 Gy. Then, for both models irradiated at 1 Gy, we studied hippocampal neurogenesis in adulthood, two to three months after irradiation. First, using BrdU injections, we compared cell proliferation in the DDG in both models, two months after postnatal irradiation. Second, we analyzed the survival and the differentiation of one population of BrdU^+^ cells one month later, at the end of a behavioral task, therefore more than three months after irradiation. Thus, we highlighted, in both models irradiated at 1 Gy, different effects of IR on adult hippocampal neurogenesis, with a more marked negative effect when targeted DDG irradiation was performed. Therefore, while investigating the effects of the irradiation dose and of the brain irradiated volume on spatial memory and adult hippocampal neurogenesis, our results underline the complexity of IR mechanisms triggered in the brain which can lead to cognitive disorders.

## 2. Materials and Methods

### 2.1. Animals

**Figure 1:**
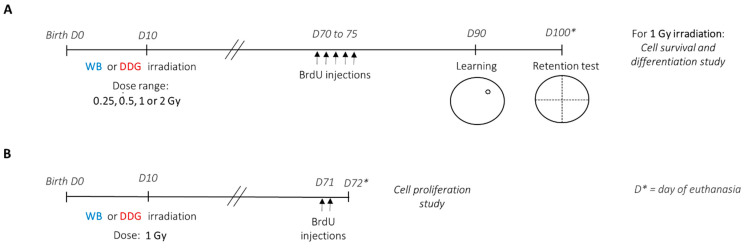
Experimental protocol. Ten-day-old C57BL6/J male mice were irradiated according to whole brain (WB) or dorsal dentate gyrus (DDG) exposure. Two months after ionizing radiation (IR), mice received BrdU injections to follow different steps of adult hippocampal neurogenesis. (**A**) In one set of experiments, mice from each group exposed to 0.25, 0.5, 1 or 2 Gy were submitted to spatial massed training at 90 days old, and the retention test was performed 10 days later, before perfusion. Brains from sham mice and mice irradiated at the dose of 1 Gy were used to analyze cell survival and differentiation of BrdU^+^ cells. (**B**) In the second set of the experiment, sham, WB and DDG irradiated mice at the dose of 1 Gy (WB_1 Gy_ and DDG_1 Gy_) were perfused 24 h after the BrdU injections to analyze cell proliferation of adult-born cells, 2 months after IR. D* = day of euthanasia.

Experimental studies detailed in Figure 1 were conducted using male progeny of C57Bl/6J mice from Charles River (L’Arbresle, France) on days 14–17 of gestation. Throughout the experimental procedure, animals were maintained under environmental controlled conditions (12:12 light-dark cycle; 20 ± 1 °C); food and water were provided ad libitum. Animal experiments were approved by the IRSN Ethics Committee no. 81 (protocol P17–12, agreement number C92-032-01).

### 2.2. Irradiation Procedure

Ten days after birth, male offspring from distinct litters were randomly subjected to different irradiation conditions. WB or targeted DDG irradiation was performed in ten-day-old anesthetized (i.p., injection of ketamine (40 mg/kg)/xylazine (0.8 mg/kg)) male mice at a single X-ray dose of 0.25, 0.5, 1 or 2 Gy (Figure 1), using the protocol described in Dos Santos et al. (2018) [17], on the Small Animal Research Platform (SARRP) (Xstrahl, Ltd., Camberley, UK).

Briefly, cone beam computed tomography (CBCT) images from the SARRP were used to irradiate the whole brain. To irradiate the DDG, CBCT images were manually superimposed to magnetic resonance images on the SARRP treatment planning system (TPS) (MuriPlan, Xstrahl, Inc., Suwanee, GA, US). For each treatment plan, two isocenters were positioned and, for each, two beams at 180° to the sagittal plane were delivered using for WB irradiation a 5 × 5 mm² collimator and for DDG irradiation a 1 mm diameter collimator. The duration of the irradiation procedure was between 15–20 min and 25–30 min, respectively, for WB and DDG irradiation. It must be highlighted that when the same dose was planified with the SARRP TPS and when different irradiation fields were used to irradiate either the DDG or the whole brain, each volume of interest received the same dose, and thus the same dose was delivered to the DDG. A voltage of 220 kV, an intensity of 3 mA and a dose rate of 0.5 Gy/min were used. Sham mice were submitted to the same procedure as the irradiated ones without being irradiated. All animals were kept warm prior to irradiation and at the exit of the SARRP.

After the irradiation procedure, anesthetized mice were tattooed on the paw pads (individual identification) and returned to their own cage when they wake up. No cannibalism and rejection from mothers were observed until weaning. After weaning and until the end of the experiments, male mice progeny were placed in groups of 5–6 individuals/cage depending on their irradiation conditions.

### 2.3. Morris Water Maze: Set-Up and Training Procedure 

To study the effects of postnatal exposure at low to moderate doses of IR on spatial memory, learning and long-term spatial memory performances were assessed three months after irradiation, using a massed-training protocol adapted from Veyrac et al., 2013 [33] (Figure 1A), in the Morris water maze. In each exposure model, the four X-ray doses administered were: 0.25, 0.5, 1 or 2 Gy. Thus, including the sham mice group, behavioral studies were carried out with nine experimental mice groups (sham *n* = 16, WB_0.25 Gy_ *n* = 9, WB_0.5 Gy_ *n* = 8, WB_1 Gy_ *n* = 12, WB_2 Gy_ *n* = 9, DDG_0.25 Gy_ *n* = 9, DDG_0.5 Gy_ *n* = 11, DDG_1 Gy_ *n* = 11 and DDG_2 Gy_ *n* = 8).

The test was conducted in a circular pool (diameter 150 cm) whose water (20 to 22 °C) was clouded with a white non-toxic opacifier (Blanc de Meudon). To record behavioral sequences, a camera connected to a video tracking system (ANY-maze™, Stoelting Europe, Dublin, Ireland) was placed above the device. Several environmental distal cues were placed all around the pool, which was divided into four virtual quadrants. An escape platform (10 cm in diameter) was placed in the center of one quadrant (i.e., the target quadrant). The three other quadrants (i.e., the starting points) were assigned pseudo-randomly and varied in every trial. The position of the hidden platform was assigned for each mouse in one of the four virtual quadrants of the maze, such that the four positions were equally used in all groups of mice. For each mouse, the platform remained at a fixed location during all training.

For habituation, each mouse performed one block of three consecutive trials, with the visible platform (0.5 cm above the water surface). The following day, mice were trained to learn the location of the hidden submerged platform (0.5 cm below the water). The massed-training protocol consisted of three training sessions separated by 2 h. Each session was composed of three blocks of three consecutive trials of 60 s (total 27 trials). Each trial had a maximum duration of 60 s. If the mouse failed to find the platform, it was guided by the experimenter’s finger toward it. The mouse remained on the platform 60 s before being placed at a new starting point for the following trial. Latency and distance to the platform, swimming speed and thigmotaxis were studied. Ten days after training, long-term spatial memory was evaluated during one probe test of 60 s without the platform. We analyzed the percent distance swum and the percent time spent in the quadrant that previously contained the platform to chance level (target quadrant, 25%).

### 2.4. 5-Bromo-2’-Deoxyuridine (BrdU) Administration

The study of different steps of adult hippocampal neurogenesis, in both models irradiated at 1 Gy (WB_1 Gy_ and DDG_1 Gy_), was carried out 2 months (cell proliferation study) and more than 3 months (cell survival and differentiation study) after irradiation (Figure 1A,B). For cell proliferation analyses, two months after irradiation, mice (sham *n* = 6, WB_1 Gy_ *n* = 6 and DDG_1 Gy_ *n* = 6) received two intraperitoneal injections of BrdU (150 mg/kg), separated by 4 h, and were euthanized 24 h after the last one (Figure 1B). To study cell survival and differentiation, two months after irradiation, mice (*n* = 6–7) received one intraperitoneal injection of BrdU (50 mg/kg) per day, during five consecutive days. BrdU administration was given 4 weeks before the beginning of the massed-training task, and mice were euthanized after the retention test (Figure 1A).

### 2.5. Brain Sample and Tissue Section

Mice were deeply anesthetized with a mix of ketamine (40 mg/kg: IMALGENE 1000 (Boehringer Ingelheim Animal Health)/xylasine (4 mg/kg: ROMPUN 0.2% (Bayer)). They were then perfused transcardially successively with two cold solutions: a saline solution (0.9% NaCl) and then with a 4% paraformaldehyde solution in PBS (pH = 7.4). Brains were dissected, post-fixed in 4% paraformaldehyde/PBS 1X for 24 h at 4 °C and then cryoprotected in a solution of 30% sucrose/PBS 1X for 24 to 48 h at 4 °C. After that, brain samples were frozen in an optimal cutting temperature compound and stored at −80 °C. Brain coronal sections of 10 μm (−1.06 mm to −1.82 mm, bregma) were sliced using a cryostat (Thermo Scientific™ Microm™ HM 550, Erlangen, Germany).

### 2.6. Immunohistofluorescence

Immunostainings were performed in brain sections of sham, WB_1 Gy_ and DDG_1 Gy_ mice. After rehydration, sections were treated with a solution of formamide (Sigma Aldrich 47670, Saint Quentin Fallavier, France): SSC2X (Gibco 15557, Illkirch, France)/50:50 for 2.5 h at 69 °C and then incubated with a solution of 1 N hydrochloric acid for 45 min at 37 °C. After 3 washes, they were incubated with a saturation solution (E17-100) to block non-specific binding for 30 min. Brain slices were then incubated overnight at 4 °C with sheep anti-BrdU–polyclonal antibodies (1:50; pab9791, abnova VWR, Fontenay-sous-Bois, France) to study cell proliferation, or in combination with either mouse anti-NeuN polyclonal antibodies (1:1000; MAB377, Millipore, Saint Quentin en Yvelines, France) or with rabbit anti-GFAP polyclonal antibodies (1:500; ZO334, Millipore, Saint Quentin en Yvelines, France ) to study cell survival and cell differentiation. After washes, the slices were incubated for 2 h at room temperature with the appropriate secondary antibodies: donkey anti-sheep Alexa fluor 594 (1:200; A11016, Millipore, Saint Quentin en Yvelines, France), goat anti-rabbit Alexa fluor 488 (1:200; A11034, Millipore) and goat anti-mouse Alexa fluor 647 (1:200; A21236, Millipore, Saint Quentin en Yvelines, France) diluted in PBS 1X/Triton 0.1%. The slices were finally mounted in Vectashield medium with DAPI (H-1200, Vector Laboratories, Inc., Burlingame, CA, USA) after several washes.

### 2.7. Cell Quantification

Quantifications were performed in the DDG granule cell layer of 12 sections per animal (*n* = 6 to 7/group) separated by 50 µm. BrdU^+^, BrdU^+^/NeuN^+^ and BrdU^+^/GFAP^+^ cells were counted manually with an Olympus microscope (BX60) coupled with mapping software (Mercator Pro, Explora Nova, La Rochelle, France) or with a confocal microscope (Carl Zeiss MicroImaging, LSM 780 NLO, Jena, Germany) at objective x20. The total surface of granule cell layers and the DDG were established by software (BX60 microscope, Mercator Pro; Explora Nova, La Rochelle, France) to obtain the densities of BrdU^+^ cells, i.e., the number of BrdU^+^ cells/µm² of surface. To obtain the percentage of BrdU^+^/NeuN^+^ and BrdU^+^/GFAP^+^ cells (38- to 43-day-old), the phenotype of at least 300 BrdU^+^ cells per group was analyzed.

### 2.8. Statistical Analysis

Data normality was checked by Q-Q plot methods. One (dose or model) or two-way (dose x model) repeated-measures ANOVA was used for intra- and inter-group analysis to study spatial learning performances. To compare the interaction dose x model, we randomly chose eight sham mice to become the respective controls for both models. One simple t-test relative to 25% was used to compare long-term memory performances against chance, during the probe test. An intergroup comparison was made using t-test if the data were distributed according to the normal law, otherwise, a Mann–Whitney was applied (Sigma^®^ v11.0). For immunochemistry data, a general equation analysis was conducted for intergroup comparisons (R version 3.4.4, R Core Team, Boston, MA, USA; version 1.1.423, RStudio). This test is based on the hypothesis that data from the same mouse are dependent and data from different animals are independent. The two-proportion Z-test was used to compare data expressed as a percentage. All data are presented as means +/− SEM; the significance level was set at *p* < 0.05.

## 3. Results

### 3.1. Postnatal Irradiation Did not Affect Learning Performances, Only Long–Term Spatial Retrieval after DDG Exposure at 1 Gy

**Figure 2:**
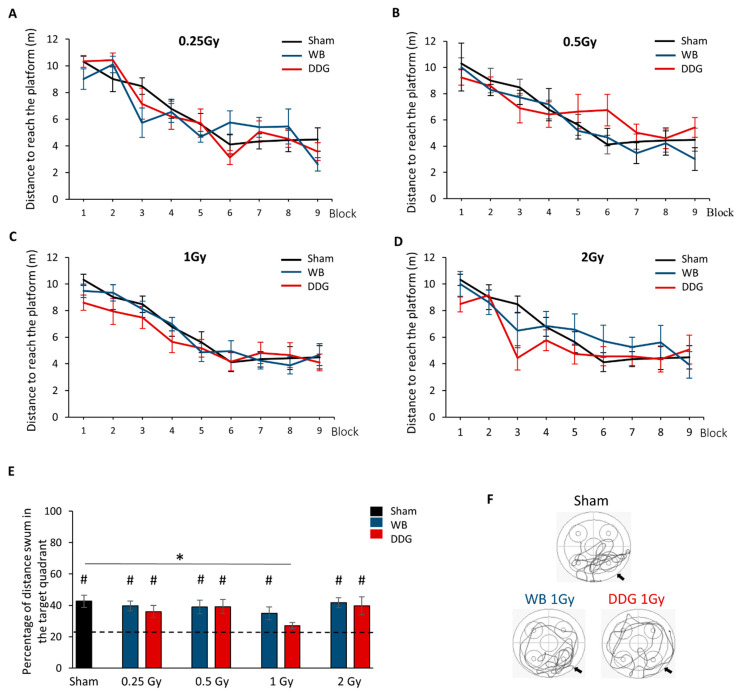
Learning and long-term spatial memory performances in the Morris water maze task, 3 months after irradiation. (**A**–**D**) Distance to reach the hidden platform during training for sham, WB and DDG irradiated mice at the doses of (**A**) 0.25 Gy, (**B**) 0.5 Gy, (**C**) 1 Gy and (**D**) 2 Gy. (**E**) Percent distance swum in the target quadrant during the probe test (# *p* < 0.05, the chance level is shown by the dotted line (25%); * *p* < 0.05). All data are expressed as means ± SEM (sham *n* = 16, WB *n* = 8 to 12 and DDG *n =* 8 to 11). (**F**) Example of trajectory during the memory test for sham, WB and DDG mice irradiated at the dose of 1 Gy. Black arrows indicate the target quadrant.

Three months after irradiation of the whole brain or DDG at doses of 0.25, 0.5, 1 or 2 Gy, we assessed spatial memory using a massed-training protocol in the Morris water maze. No impairment of the learning process was found in the two models of exposure at each dose as shown by the significant decrease in distance swum to reach the hidden platform (block effect for each group, *p* < 0.001, see statistical details in Table 1, Figure 2A–D). For each model studied independently, no dose effect was observed in the learning process (WB exposure: dose effect, *ns* and block × dose interaction, *ns*; DDG exposure: dose effect, *ns*; block × dose interaction, *ns*, see statistical details in Table 2). The comparison between models, for each dose, did not reveal significant differences (for each dose: model effect, *ns*; block × model interaction, *ns*, see statistical details in Table 3). Finally, no dose × model interaction was found (*ns*). Thus, no impairment of spatial learning was observed three months after irradiation, whatever the exposure dose and the postnatal irradiation model (WB *versus*. DDG). To complete, swim speeds and thigmotaxis (distance swum in periphery) were similar during training (data not shown), revealing the absence of motor limitations and exacerbated anxiety in our conditions.

During the probe test performed 10 days after training, the percent distance swum by sham mice in the target quadrant (42.62%) or percent of time spent in target quadrant (42.55%) were significantly different from the 25% chance level (respectively, one-sample *t* test: *t* = 4.393, *p* <0.001, *t* = 4.419, *p* <0.001, Figure 2E). Concerning irradiated WB and DDG mice, no spatial memory deficit was found at 0.25 Gy and 0.5 Gy (respectively, % distance in target quadrant: *p*_WB_ < 0.002; *p*_DDG_ = 0.025; *p*_WB_ = 0.02; *p*_DDG_ < 0.012; % time spent in target quadrant: *p*_WB_ = 0.006; *p*_DDG_ = 0.048; *p*_WB_ = 0.031; *p*_DDG_ = 0.01). In contrast, at 1 Gy, mice did not swim significantly more or not spend significantly more time in the target quadrant after DDG targeted irradiation, indicating an impairment of long-term spatial memory (% distance: 27.10%, *t* = 0.992, *p* = 0.345; % time in target quadrant: 26.77%, *t* = 0.660; *p* = 0.524) but not after WB exposure to IR (% distance: 34.93%, *t* = 2.381, *p* = 0.036, % time in target quadrant: 35.40%, *t* = 2.225; *p* = 0.048; Figure 2E,F). Moreover, the distance swum or time spent in the target quadrant significantly differed between sham and DDG_1 Gy_ mice (Figure 2E, Mann–Whitney test, % distance: U = 30, *p* = 0.005; *t*-test, % time: *t* = 2.976, *p* = 0.006).

For both models, 2 Gy exposure did not impact spatial memory as revealed by percent distance swum or time spent in the target quadrant compared to chance level (% distance swum: WB_2 Gy_: 41.67%, *t* = 4.931, *p* = 0.001 and DDG_2 Gy_: 40.82%, *t* = 2.400, *p* = 0.047; % time spent: WB_2 Gy_: 43.25%, *t* = 4.359, *p* = 0.002 and DDG_2 Gy_: 43.52%, *t* = 2.265, *p* = 0.037). Taken together, these results suggest that juvenile X-ray DDG targeted exposure at the dose of 1 Gy impaired long-term spatial memory three months later, in contrast to WB irradiation. Moreover, our data highlighted a non-monotonic dose-effect relationship in adult mice after DDG targeted postnatal irradiation.

### 3.2. Postnatal Irradiation at 1 Gy Impacted Adult Dentate Gyrus Cell Proliferation Differently in the Two Models

**Figure 3:**
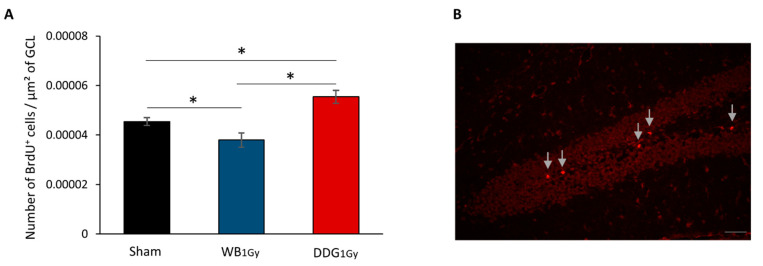
Cell proliferation, 2 months after irradiation at the dose of 1 Gy. (**A**) Density of BrdU^+^ cells in the granule cells layer (GCL) of sham, WB_1 Gy_ and DDG_1 Gy_ irradiated mice. Data are reported as mean ± SEM (*n* = 6/group, * *p* < 0.05). (**B**) Photomicrograph showing BrdU staining in the GCL (scale bar = 50 µm).

Given our behavioral results, we focused our adult hippocampal neurogenesis analysis after postnatal irradiation at 1 Gy in both models. Cell proliferation was studied two months after irradiation, by analyzing the density of BrdU^+^ cells in the granule cells layer (GCL) of sham, WB_1 Gy_ and DDG_1 Gy_ irradiated mice, 24 h after BrdU injections (Figure 3A,B). Our results show that WB_1 Gy_ postnatal irradiation significantly decreased the density of proliferating cells compared to sham mice (*p* = 0.0292). In contrast, DDG_1 Gy_ exposure significantly increased the density of proliferating cells (*p* = 0.0034). A significant difference was also found between the two models of exposure exposed at 1 Gy (*p* < 0.0001). Thus, our data demonstrated an opposite effect of IR on dorsal DG cell proliferation in the two models exposed at 1 Gy, two months after postnatal exposure.

### 3.3. Postnatal Irradiation at 1 Gy Impacted Differentiation Only after DDG Targeted Exposure

**Figure 4:**
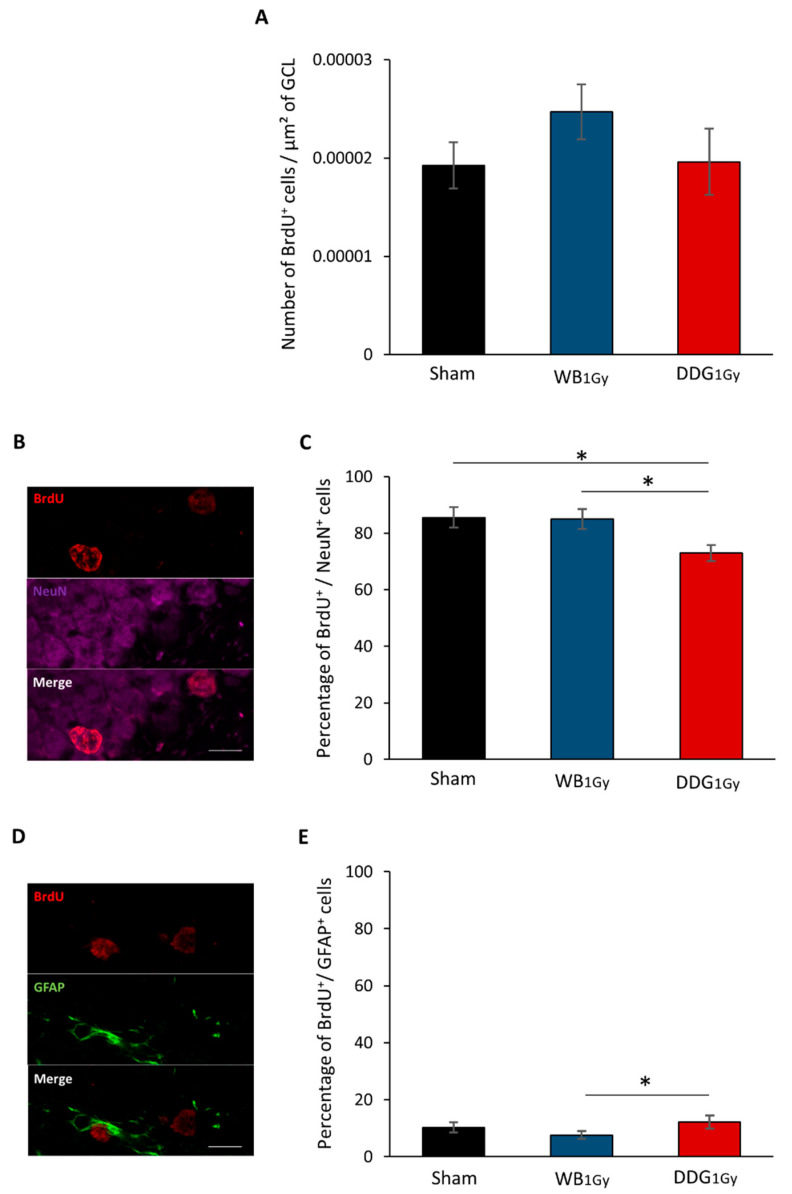
Newborn cell survival and differentiation studied after the retention test, 3 months and 10 days after irradiation at the dose of 1 Gy. (**A**) Density of BrdU^+^ cells in the GCL of sham, WB_1 Gy_ and DDG_1 Gy_ irradiated mice. (**B**) Confocal photomicrograph of BrdU^+^ cells expressing NeuN (scale bar = 10 µm). (**C**) Percentage of BrdU^+^/NeuN^+^ cells in the GCL of sham, WB_1 Gy_ and DDG_1 Gy_ irradiated mice. (**D**) Confocal photomicrograph of BrdU^+^ cell expressing GFAP (scale bar: 10 µm). (**E**) Percentage of BrdU^+^/GFAP^+^ cells in the GCL of sham, WB_1 Gy_ and DDG_1 Gy_ irradiated mice. All data are expressed as mean ±SEM (*n* = 6 to 7/group, * *p* < 0.05).

The density of BrdU^+^ cells analyzed five to six weeks after BrdU injections in the DDG was similar between groups, which revealed no deficit in the density of newborn cells, three months and 10 days after exposure (*p*_Sham/WB1 Gy_ = 0.51; *p*_Sham/DDG1 Gy_ = 0.53; *p*_WB1 Gy /DDG1 Gy_ = 0.22, Figure 4A). Nevertheless, when we studied the phenotype of these cells, the percentage of BrdU^+^ cells co-expressing NeuN in the GCL (Figure 4B) was significantly decreased in DDG_1 Gy_ irradiated mice compared to sham (∆ = −12.64%, *p* = 0.003) and to WB_1 Gy_ irradiated mice (∆ = −12.07%, *p* < 0.001). No significant difference was found between WB_1 Gy_ irradiated and sham mice (*p* = 0.8242, Figure 4B,C). Moreover, DDG targeted exposure at 1 Gy significantly increased the percentage of BrdU^+^ cells co-expressing astroglial marker (GFAP) compared to WB_1 Gy_ irradiated mice (*p* = 0.0028), but not compared to sham mice (*p* = 0.1784). The percentage of cells co-expressing BrdU and GFAP remained unchanged after WB_1 Gy_ exposure compared to sham mice (*p* = 0.3267, Figure 4D,E). In conclusion, 1 Gy irradiation of 10-day-old mice impacted some steps of adult hippocampal neurogenesis and resulted in defective differentiation of adult-born neurons, more than three months later, only in DDG_1 Gy_ irradiated mice.

Finally, our results underline a major effect of irradiation volume in the disturbance of adult hippocampal neurogenesis observed three months after postnatal exposure to IR at a dose of 1 Gy.

## 4. Discussion

Until now, very few studies have investigated the impact of low to moderate doses of IR on the postnatal brain and their potential consequences for cognitive processes in adulthood. In the present work, we report no spatial learning and memory deficit after postnatal WB exposure to IR (≤2 Gy) whereas when irradiation beams were focused on the DDG, adult mice displayed altered spatial memory restitution, without impairing their learning, at 1 Gy, but not at the other tested doses. Finally, these contrasting effects observed in both models (WB/DDG) at the dose of 1 Gy have been confirmed by studying adult hippocampal neurogenesis. 

### 4.1. Long-Term Spatial Memory Is Not Impacted in Adult Mice Whole Brain Postnatally Exposed to Ionizing Irradiation

Some deleterious effects of postnatal IR (≤2 Gy) on cognitive functions in adulthood have been demonstrated in a few studies in the literature. For instance, some authors have revealed alterations in spontaneous behaviors (locomotion/general activity/rearing) of adult mice exposed to a novel environment several months after whole body exposure, from the dose of 0.35 Gy, on postnatal day 10 (PND 10) [14,15,16]. We have also previously shown higher anxiety/depressive-like behaviors in adult mice after postnatal brain exposure (PND 10; from 0.25 to 1 Gy; [17]). In contrast, while some spontaneous behaviors seem to be modified, other cognitive functions such as spatial working memory tested in a radial arm maze 1.5 months after postnatal whole body exposure (PND 10, 1 Gy; [13]) or spatial memory assessed using a distributed protocol in the water maze task (5 trials/day during three consecutive days, retention test performed on the third acquisition day) in adulthood after postnatal brain irradiation (PND10, 0.1 and 2 Gy; [18]) did not appear to be altered. In the present study, using a more stringent protocol in the water maze task in which mice have to perform 27 trials on the same day and their retention test 10 days later, we confirmed the absence of spatial learning and memory impairments after WB exposure, for all doses tested.

The different effects of IR observed in behavioral studies can be related to the type of cognitive process assessed: different kinds of memory/spontaneous or emotional behaviors. They are also probably linked to the irradiation protocol employed: whole body or whole brain. For instance, after partial whole body irradiation, even if the brain is not directly irradiated, acute systemic inflammation can promote the entry of pro-inflammatory cytokines into the brain [35]. This type of phenomenon can favor the establishment of distinct inflammatory processes after WB or whole body irradiation, which have been described after postnatal exposure (PND10; ≤2 Gy [15,18]) and, finally, help explain the behavioral differences observed.

### 4.2. Dose and Brain Irradiated Volume Effects on Long-Term Spatial Memory in Adult Mice Postnatally Exposed to Ionizing Radiation: A Complex Response

Here, we showed, for the first time, a deficit in spatial memory retrieval, despite normal learning, only in adult mice whose DDG was irradiated at 1 Gy at PND 10. Thus, at the dose of 1 Gy, millimetric targeted DDG irradiation appears to be more deleterious to spatial memory than WB irradiation in our conditions. This result highlights, in an unexpected way, the irradiated volume effect, this time in the brain, in mechanisms leading to radiation-induced cognitive disorders. Interestingly, the data yielded by our DDG model indicated a non-monotonic dose-response relationship, with a retrieval deficit after exposure at 1 Gy but not after lower (0.25 and 0.5 Gy) or higher (2 Gy) IR doses. To our knowledge, such a dose-effect relationship in behavioral studies has been observed only once, when assessing short-term memory a few hours after whole body exposure, for doses of 2, 5 and 8 Gy in adult female mice [36]. This intriguing result is not in accordance with the relationship between hippocampal dose level and the risk of developing neurocognitive impairment, described at high doses in radiotherapy [22] (for review, see [37]), and needs to be further investigated for a better understanding in our dose-range study.

In the present work, the specific action mechanisms of IR underlying non-monotonic dose-response after DDG targeted exposure and different effects in spatial memory at the exposure dose of 1 Gy, in both models, could be explained by several biological processes. For instance, opposing/compensatory effects (redox balance and inflammation) may or may not appear, depending on the volume irradiated and the irradiation dose, and thus participate in the different responses obtained.

### 4.3. One-Gray Postnatal Irradiation of the DDG/Whole Brain Leads to Contrasting Disturbances of Adult Hippocampal Neurogenesis

Guided by the evidence that spatial memory deficits are linked to alterations in adult hippocampal neurogenesis (for reviews, see [27,28,29]), we studied the impact of IR (1 Gy) on proliferation and differentiation of newborn cells in the DG in both models. Postnatal WB exposure led to decreased cell proliferation in the DG two months after exposure, in agreement with previous work [15,18], without impacting BrdU^+^ cell density or differentiation more than one month later. However, our present data, focused on one BrdU^+^ cell population, cannot be extrapolated to a decrease in all mature granule cells in the DG, as previously demonstrated after postnatal whole body or WB exposure, respectively at doses of 1 and 2 Gy [15,18]. In contrast, two months after DDG exposure, an increase in proliferative cells was observed. However, as after WB exposure, no difference was observed in the number of five-to-six-week-old newborn cells, suggesting that one part of this population of proliferative cells did not survive after five to six weeks. Moreover, as suggested by the respective decrease and relative increase in five-to-six-week-old cells co-expressing BrdU and NeuN or BrdU and GFAP, some cells failed to differentiate into neurons and could either differentiate into mature astrocytes or stayed blocked in a progenitor state (type 1 and 2a cells). Even if we could suspect an increase in astrocytes, our data do not allow us to draw a firm conclusion on this point. Nevertheless, whether those cells were astrocytes or senescent-blocked progenitor cells, this could suggest in both cases a higher level of neuro-inflammation in our DDG model several weeks after irradiation.

The mechanisms underlying how postnatal IR of the whole brain or DDG impacts adult hippocampal neurogenesis differently have not been investigated. In the literature, it is well known that pathological conditions, such as inflammation, can modify DG cell proliferation and disturb neuronal-astrocyte production rate in favor of astrocytes [38,39]. Moreover, in the field of IR at high dose, chronic neuro-inflammation has already been observed in adult mice after IR [40], and its involvement in adult hippocampal neurogenesis disturbance has been demonstrated [41]. After postnatal irradiation, cellular and molecular changes in hippocampal mediators involved in neuro-inflammation have been described and are dose-, irradiated region- and time-dependent (PND10; ≤2 Gy [15,18]). Moreover, WB postnatal irradiation leads to blood-brain barrier dysfunction (PND 10; 0.1 and 2 Gy; [42]), probably in a very different way than DDG targeted exposure. Thus, we could hypothesize that the difference observed in the adult hippocampal neurogenesis process in the two models may also be due to radiation-induced distinct inflammatory profiles. Thus, astrocyte and microglial cell activation as immune myeloid cell infiltration will be the subject of future studies in our models.

Then, even though the current state of our results does not yet allow us to understand, in depth, the alterations in adult neurogenesis in our DDG model, we can, however, suggest that the decrease in mature granule cells contributes, at less partly, to the observed spatial memory impairments. Indeed, several studies have shown that the reduction of adult-born neurons and/or defects in their integration into brain networks leads to deficits in spatial memory [32,34,43,44]. Moreover, given the involvement of adult-born neurons of the DG in other cognitive functions, such as pattern separation function (for reviews, see [45]) or management of interference [40], further studies will be required to explore the potential impact of postnatal IR (≤2 Gy) on these functions. Finally, we cannot rule out the fact that spatial memory deficit observed after DDG irradiation at 1 Gy may also be the consequence of potential radiation-induced alterations in the physiology and/or functioning of microvessels, glial and neuronal cells [15,18], either inside the dentate gyrus or located in other surrounding brain regions. For instance, a part of CA3 of the hippocampus or cortical sensory areas are also crossed by the millimeter irradiation beams during DDG targeted exposure procedure [17] which could also have impacted the establishment of neuronal networks and their functioning and in particular modify the LTP/LTD synaptic processes, several months later.

## 5. Conclusions

Our results confirm the absence of spatial memory deficit in adulthood after postnatal WB exposure to IR (≤2 Gy) in mouse models. Nevertheless, for the first time, we highlighted a deleterious effect of a moderate dose of IR, applied during a postnatal period, in some condition, on spatial memory. More generally, our work showed that mechanisms leading to radiation-induced spatial memory disorders after exposure (≤2 Gy) of juvenile mice are complex and depend on several factors, as the irradiation dose and the brain volume irradiated. This highlights the interest in using our two irradiation models as a tool to elucidate the mechanisms of the brain’s response to IR. 

Finally, as rodents are known as more resistant to radiation than humans, it is difficult to directly compare the doses used in our experimental studies in mouse models to those used in children. However, our data shed new light on how IR, in a low/moderate doses range, can impact the developing brain, in the long term, and provide further input for the radiological protection system in children. Combined with data from existing and future experimental and epidemiological studies, they w a better assessment of the potential risk of children’s brain exposure at low doses of IR.

## Figures and Tables

**Table 1:** Statistical results obtained by repeated-measures ANOVA for the distance swum to find the platform over blocks during training, for each dose and each model.

Dose (Gy)	Group	*n*	Distance (Block Effect)
0 Gy	Sham	16	F_(8,120)_ = 11.17
			*p* < 0.0001
0.25 Gy	WB	9	F_(8,64)_ = 8.68
			*p <* 0.0001
	DDG	9	F_(8,64)_ = 10.70
			*p <* 0.0001
0.5 Gy	WB	8	F_(8,56)_ = 6.79
			*p* < 0.0001
	DDG	11	F_(8,80)_ = 3.30
			*p* < 0.0001
1 Gy	WB	12	F_(8,88)_ = 11.50
			*p* < 0.0001
	DDG	11	F_(8,80)_ = 6.48
			*p* < 0.0001
2 Gy	WB	9	F_(8,64)_ = 4.98
			*p* < 0.0001
	DDG	8	F_(8,56)_ = 5.48
			*p* < 0.0001

**Table 2:** Statistical results for the one-way (dose effect) ANOVA with repeated measures for the distance swum to reach the platform, for the WB and the DDG irradiation model.

Models	Block	Dose	Block X Dose Interaction
WB	F_(8,392)_ = 35.288	F_(4,49)_ = 0.172	F_(32,392)_ = 0.956
	*p* < 0.0001	*p* = 0.952, *ns*	*p* = 0.809, *ns*
DDG	F_(8,400)_ = 29.087	F_(4,50)_ = 0.738	F_(32,400)_ = 1.114
	*p* < 0.0001	*p* = 0.570, *ns*	*p* = 0.465, *ns*

**Table 3:** Statistical results for the one-way (model effect) ANOVA with repeated measures for the distance swum to reach the platform, for each dose.

Dose	Block	Model	Block X Model Interaction
0.25 Gy	F_(8,248)_ = 25.061	F_(2,31)_ = 0.107	F_(16,248)_ = 1.311
	*p* < 0.0001	*p* = 0.899, *ns*	*p* = 0.191, *ns*
0.5 Gy	F_(8,256)_ = 17.564	F_(2,32)_ = 0.448	F_(16,256)_ = 0.892
	*p* < 0.0001	*p* = 0.643, *ns*	*p* = 0.579, *ns*
1 Gy	F_(8,288)_ = 26.148	F_(2,36)_ = 0.590	F_(16,288)_ = 0.487
	*p* < 0.0001	*p* = 0.560, *ns*	*p* = 0.953, *ns*
2 Gy	F_(8,240)_ = 15.471	F_(2,30)_ = 0.596	F_(16,240)_ = 1.217
	*p* < 0.0001	*p* = 0.557, *ns*	*p* = 0.3825, *ns*

## Data Availability

The raw data that support the findings of this study are available from the corresponding authors, (Drs Christelle Durand / Roseline Poirier), upon reasonable request.
